# Jaffe-Campanacci syndrome; a case series and review of the literature

**DOI:** 10.1186/s12891-024-07581-0

**Published:** 2024-06-27

**Authors:** Ahmed O. Sabry, Ahmed Salem Abolenain, Noureldin Mostafa, Abdelraouf Ramadan, Mohamed Ghanem

**Affiliations:** Department of Orthopedics, Faculty of Medicine, Cairo University, Cairo, Egypt

**Keywords:** Jaffe–Campanacci syndrome, Neurofibromatosis type 1, Café au lait macules, Non-ossifying fibroma, Case report

## Abstract

**Background:**

Jaffe-Campanacci syndrome is a rare syndrome, characterized by multiple non-ossifying fibromas (NOF) and cafe-au-lait patches. The name was coined in 1982 by Mirra after Jaffe who first described the case in 1958. Although it’s suggested there is a relation with Neurofibromatosis type 1, there is still no consensus on whether Jaffe-Campanacci syndrome is a subtype or variant of neurofibromatosis-1(NF-1).

**Case presentation:**

In this article, we present a case series of 2 patients. The first case is a 13-year-old male with Jaffe-Campanacci syndrome who presented with a distal femur fracture. His father had positive features of both Jaffe-Campanacci syndrome and NF-1, while his sister only had features of NF-1, so we presented both.

**Conclusion:**

Jaffe-Campanacci has a clear relationship with type 1 neurofibromatosis, which still has to be genetically established. Due to the presence of several large non-ossifying fibromas of the long bones, it is linked to a significant risk of pathological fractures. We concur with previous authors, that an osseous screening program should be performed for all patients with newly diagnosed type 1 neurofibromatosis, to identify non-ossifying fibromas and assess the potential for pathological fracture. Moreover, siblings of patients with NF-1 should be screened for multiple NOFs that may carry a high risk of pathological fractures.

## Background

Jaffe-Campanacci syndrome (JCS) was first demonstrated in the literature in 1958 [1]. Since then less than 30 cases have been reported [2]. JCS generally presents with café-au-lait macules, central giant cell granulomas of the jaw, and multiple non-ossifying fibromas. Other features include cardiovascular malformations, mental retardation, cryptorchidism, and hypogonadism [3].

## Case presentation

### First case

A 12-year-old Egyptian boy presented to our emergency room with acute right distal femoral pain, swelling, and an inability to bear weight, after falling to the ground earlier on the same day.

**Fig. 1:**
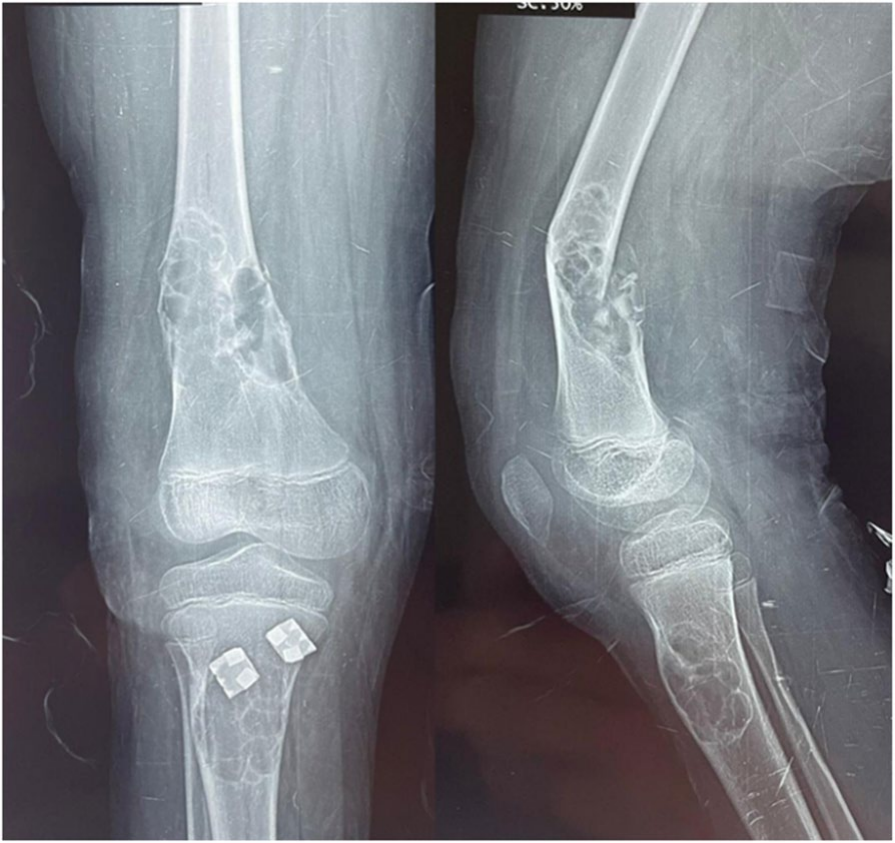
Showing an X-ray of the right femur fracture over the NOFs. A lesion in the proximal tibia is also seen

Upon examination, there was marked tenderness, ecchymosis, and deformity over the right distal femur with an inability to move his knee due to pain. X-rays showed a fractured right distal femur over a bone lesion that’s multiloculated, radiolucent with sclerotic rims consistent with a non-ossifying fibroma (NOF). The radiographs also showed a similar lesion in the proximal tibia on the right side. A full skeletal survey was done and a similar lesion was discovered in the left distal femur (Fig. 1).

**Fig. 2:**
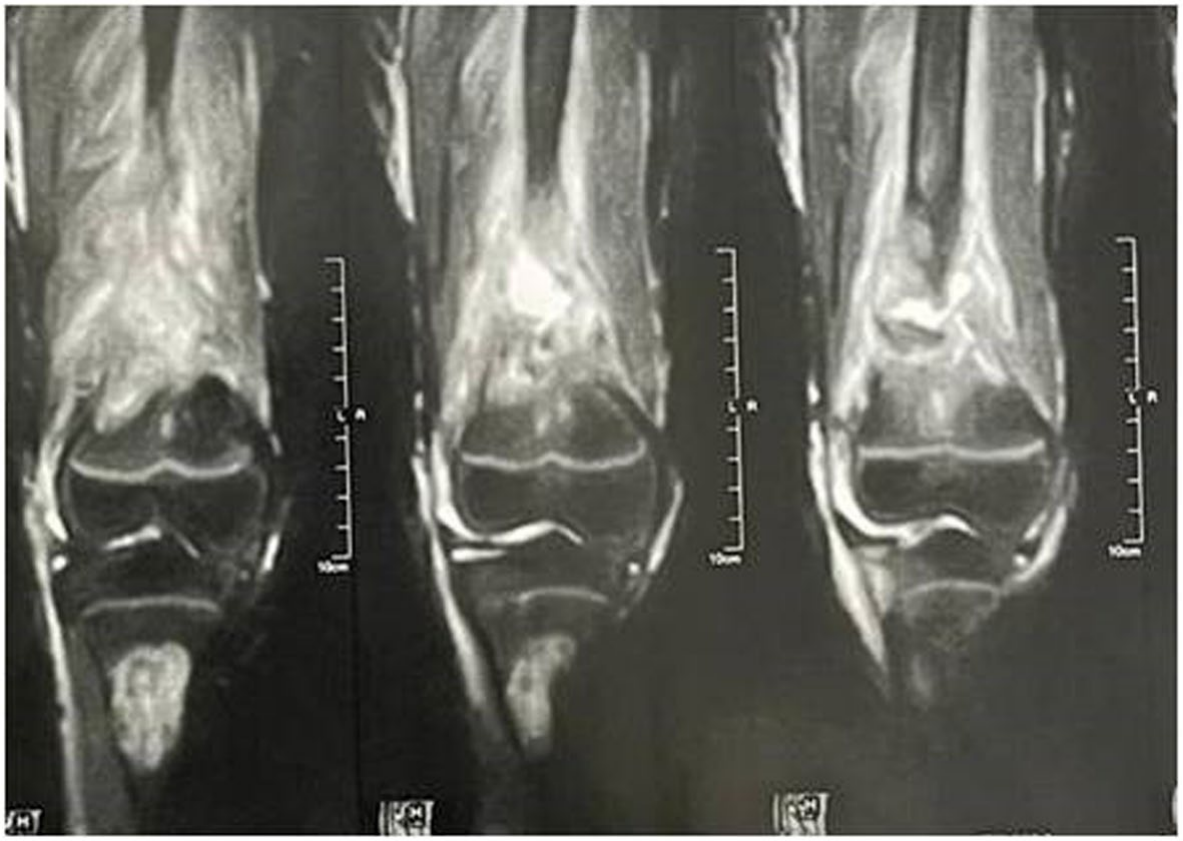
MRI of the NOF in the distal femur and proximal tibia. The Distal femur is surrounded by effusion due to the fracture

No lesions of the vertebral bodies or mandible were detected. MRI of the knees showed an eccentric, well-defined, metaphyseal, cortico-medullary lesion that causes cortical thinning with no soft tissue invasion. The lesion has a low intensity on T1 and an intermediate T2 signal, with a peripheral low signal rim corresponding to the sclerotic border (Fig. 2).

**Fig. 3:**
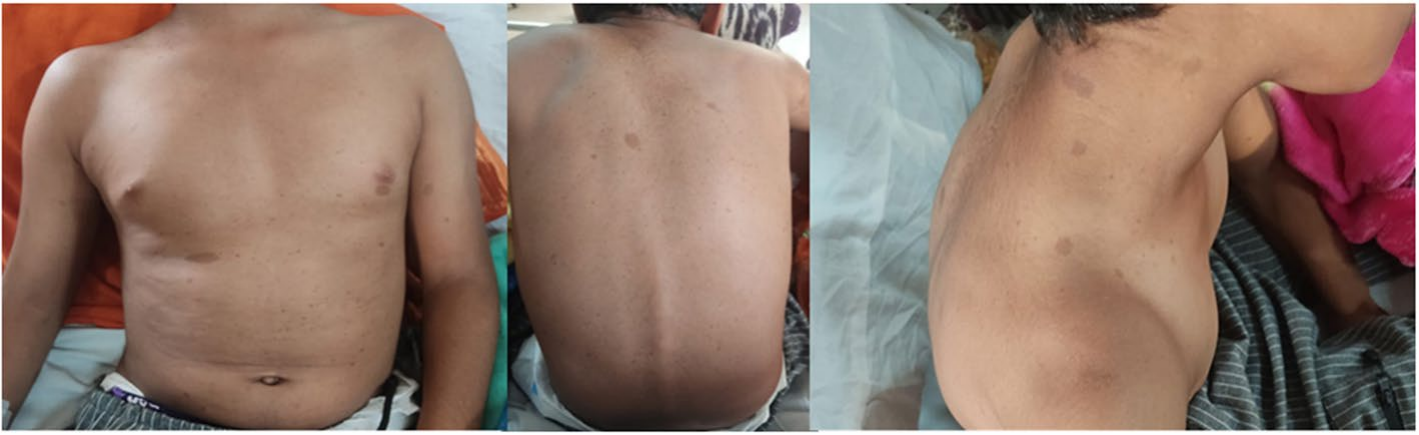
Photo shows scattered café-au-lait macules over the boys body

Upon further examination of the body, about 20 café au lait macules were scattered over his face, chest, abdomen, back, and limbs, with the highest density around the back and abdomen (Fig. 3).

The boy had multiple axillary, inguinal freckles, a short stature being below the 5th percentile for his age, a relatively large head, and suffered from hypogonadism, thus the patient was diagnosed to have JCS. No cutaneous neurofibromas or lisch nodules were detected. Ophthalmologic examination showed no significant findings.

### Management

**Fig. 4:**
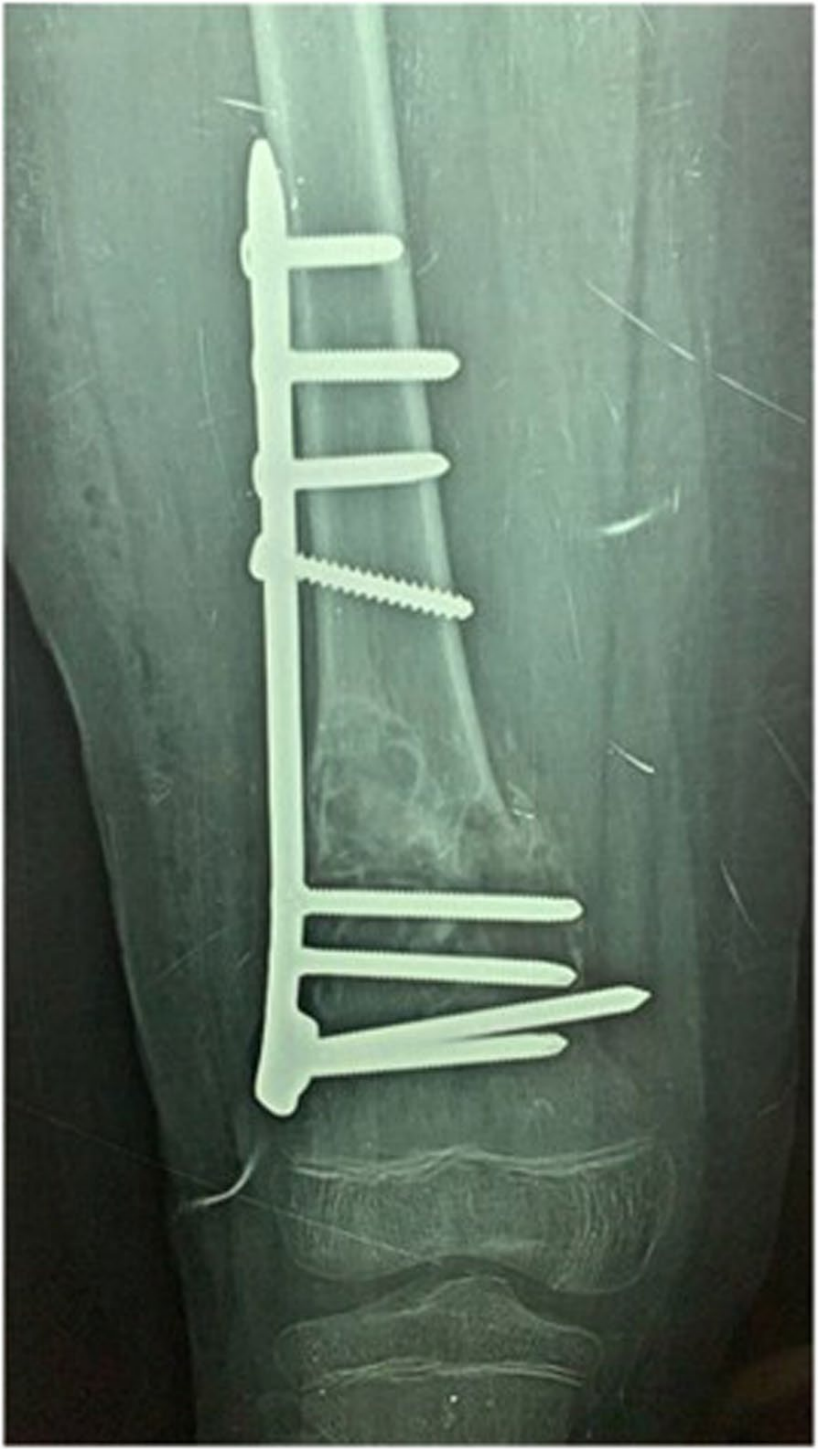
Post-operative xray after reduction and fixation of the pathological fracture

**Fig. 5:**
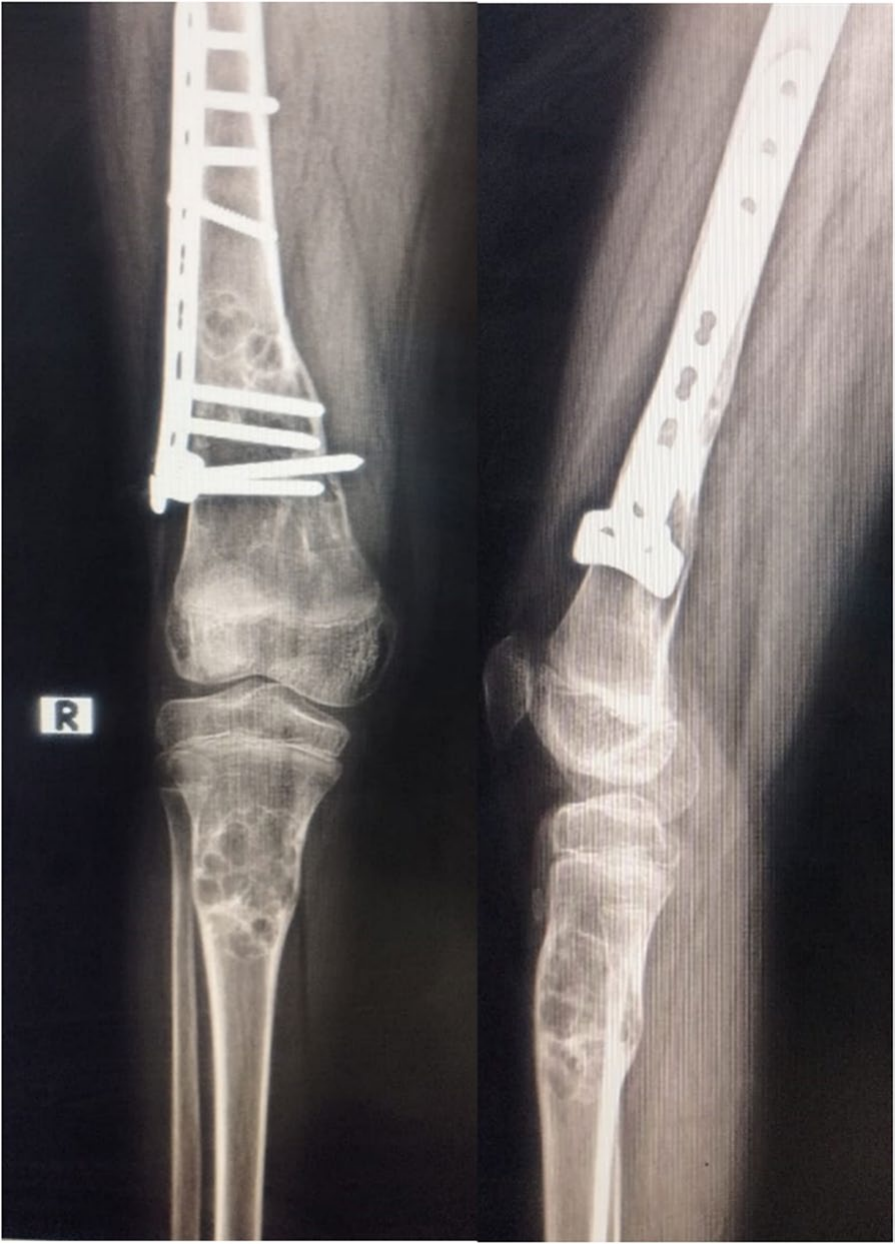
Postoperative xray showing union and calcification of the NOF’s

Skin traction was done in the emergency room and the patient was admitted to the ward for preparation for surgery of open reduction and internal fixation of the distal femoral fracture. The patient was taken into the preoperative holding area where he was side-marked. He was later moved into the operative room. Second generation cephalosporins were given to the patient IV according to pre-operative protocol. General anesthesia was administered smoothly via an endotracheal tube without complications. He was placed in a lateral position on his right side. A straight 7 cm incision was done over the lateral aspect of the thigh with dissection of subcutaneous tissues, then an incision through the iliotibial band was done. The vastus lateralis muscle was elevated from the distal femur using two bone levers, the fracture site was exposed and reduction was done by traction and fracture manipulation, fixation by a locking T-plate was done and placed above the physeal growth plate, the wound was irrigated with saline and a drainage tube was placed, closure was performed in anatomical layers the iliotibial band was sutured using vicryl 0 then the subcutaneous layer was closed with vicryl 2/0 running sutures and finally the skin was closed using monocryl 3/0 subcuticular sutures. A sterile dressing was placed over the sutured incision. An X-ray was done after the operation (Fig. 4) the drain was removed after 2 days, and the patient was discharged. The patient started protected weight bearing after 6 weeks and follow-up x-rays showed union on the 12th week where he started full weight bearing (Fig. 5).

### Second case

**Fig. 6:**
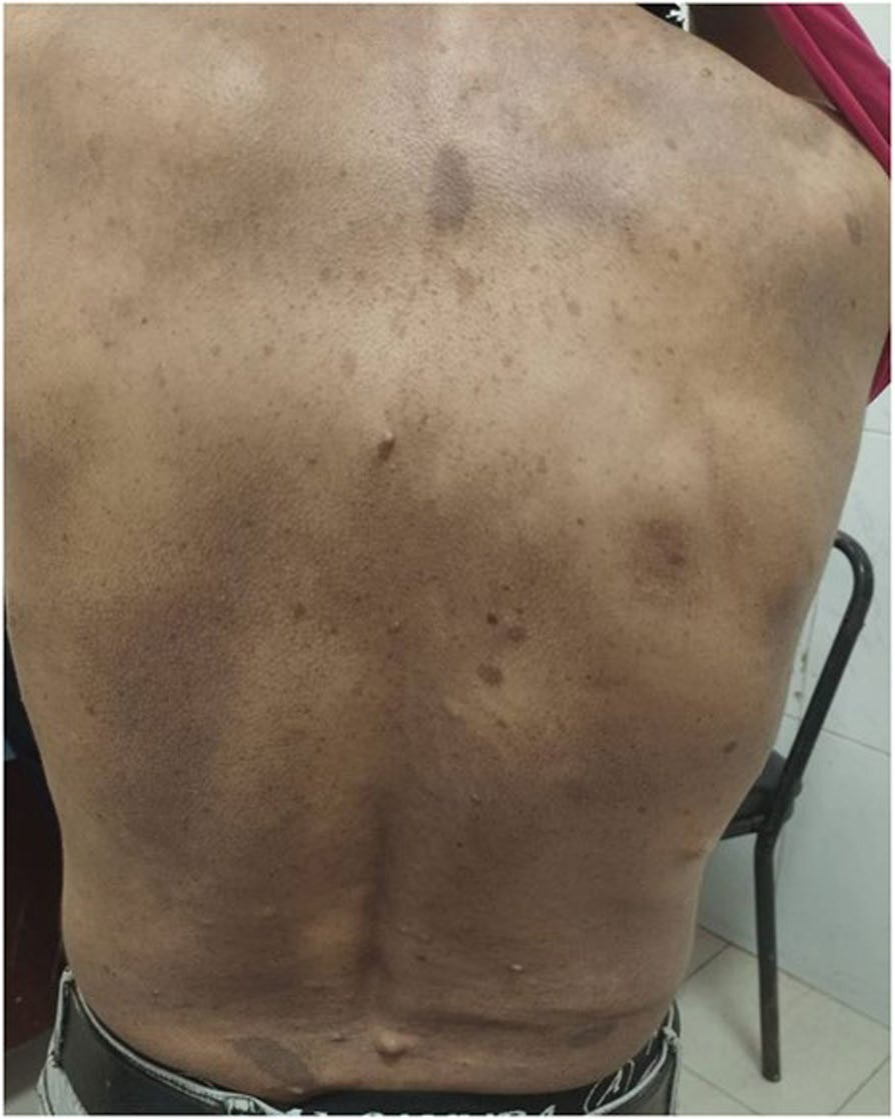
Photo of the fathers back with multiple café-au-lait macules and neurofibromas

The boy's father, a 32-year-old gentleman, upon general examination, showed almost 30 cafe au lait macules over his body, axillary and inguinal freckles, and multiple cutaneous neurofibromas spread over his body, nape, and face (Fig. 6).

**Fig. 7:**
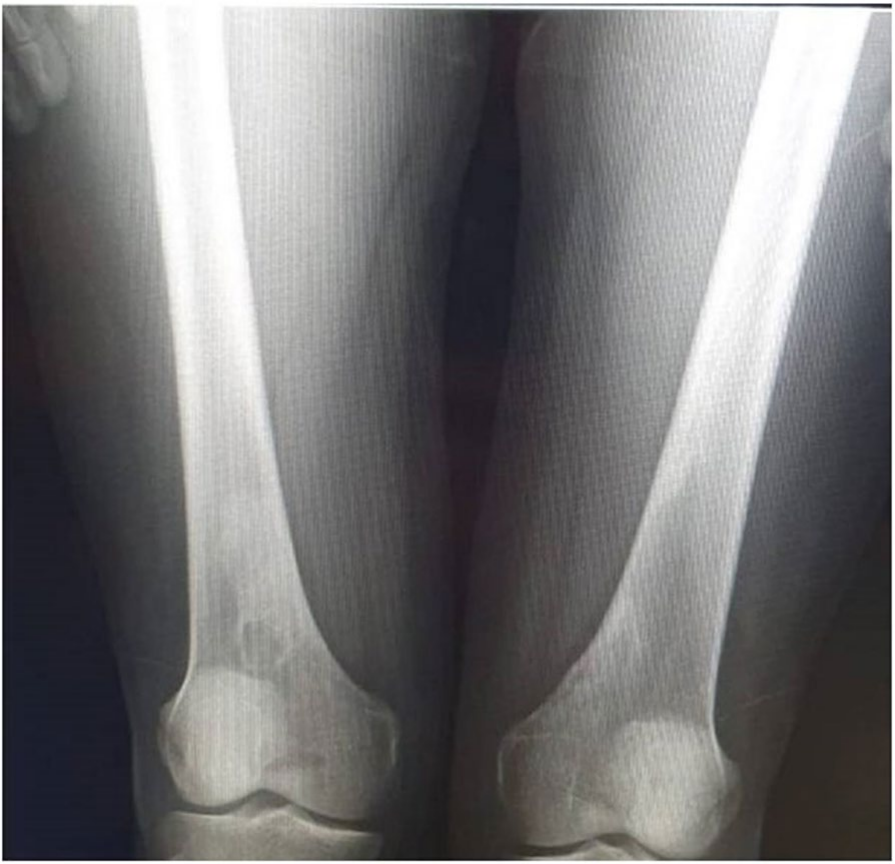
X-ray of the father shows healed NOF in the right and left distal femur

He was short in stature. Ophthalmologic examination showed no significant findings. A skeletal survey showed healed calcified non-ossifying fibromas in the distal femur of both knees (Fig. 7).

He gave a history of having a Ventricular septal defect to which he underwent surgical repair when he was 7 years old and didn’t have a history of past fractures. The father's skeletal survey showed residues of healed non-ossifying fibromas. Further history revealed the father saying his mother had similar dark-colored skin lesions and bumps. One of his sisters and a brother out of his 5 siblings also had similar skin lesions. He said his brother who had these skin lesions suffered from a fracture when he was young from a trivial fall, suggesting it could have been a pathological fracture, but we couldn’t obtain any imaging. The father said he had another 4 step-brothers from his father and none of them had these lesions, suggesting that the syndrome could have been inherited from his biological mother.

### Third case

**Fig. 8:**
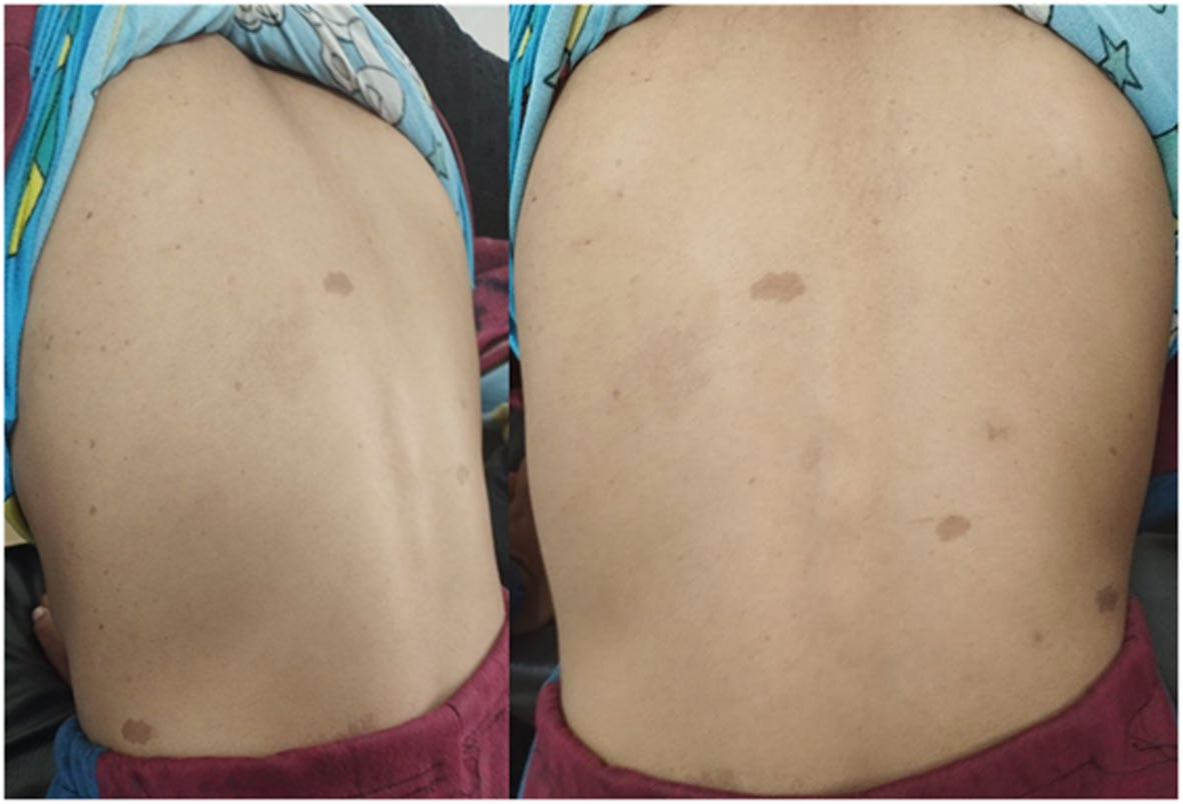
Showing café-au-lait macules

The boy's sister from the affected father, a 9-year-old girl, also showed multiple Café-au-lait macules (CALMs) over her body and axillary freckling (Fig. 8). She had a single subcutaneous neurofibroma over her neck. She had short stature compared to her age with mild cognitive disability.

Interestingly, her skeletal survey showed no non-ossifying fibromas, hence she couldn’t be diagnosed with JCS.

### Consent to publish

The patients and their guardians (Both father and mother) gave their consent for publication of their case study and scientific dissemination of the non-identifiable clinical and radiological images as a case report to medical literature in an open access journal, in the form of informed and written consent.

## Discussion

### Clinical presentation

To our knowledge, this is the first case of JCS recorded in Egypt and the second in the Middle East following a case reported in Qatar in 2005 [4].

The prevalence of non-ossifying fibroma is still unknown, as the majority of cases are asymptomatic; however, the estimated percentage is 30 to 40% of children and adolescents according to the 2020 WHO classification of tumors of the bone [5]. On the other hand, multiple NOFs are much less common, and their association with NF-1 is infrequent. Moser et al. found only 72 cases (8%) with multiple NOFs in a series of 900 patients with NOFs, from which only 4 cases (0.4%) were associated with NF-1 [6].

Non-ossifying fibromas are mainly diagnosed based on radiographic findings. On X-rays, they present as multiloculated, lucent lesions with a thin sclerotic rim, eccentrically located in the metaphysis near the physis. In MRI, NOFs exhibit a high or intermediate T2 signal, with a peripheral low signal rim corresponding to the sclerotic border. As NOFs mature, they may calcify, leading to opacity within the lesion. In MRI, the signal becomes lower on both T1 and T2 sequences [5, 7].

### Diagnostic challenges

JCS can be differentiated from NF-1 when spotting multiple non-ossifying fibromas with multiple café-au-lait macules in the absence of cutaneous or nervous neurofibromas [1, 3] However, several authors have studied the overlap between JCS and neurofibromatosis type 1 suggesting that JCS is a special subtype of NF-1 [3]. Our first case has signs suggesting JCS as described by Mirra et al. being presented with a pathological fracture, with some signs of NF-1 and being stunted with average height below the 5th percentile for his age and sex and also hormonal disturbance in the form of hypogonadism [8]. The second case is the father of the first case who was found to have multiple CALMs, axillary freckles, stunted growth multiple cutaneous neurofibromas, and healed NOFs which suggests the inheritance of NF-1 gene sequence from father to son. The detailed history of the second case revealed a mother (who is a grandparent of the first case) who had CALMs and cutaneous neurofibromas, and four siblings with CALMS suggesting that we have a family with different variants of NF-1 one of them is our first case with JCS. The third case is the sister of the first case and the daughter of the second case. She shows multiple CALMs and axillary freckles, mental retardation, and stunted growth, however, she has a single cutaneous plexiform neurofibroma and a free skeletal survey. The previous pattern of case series hints at the autosomal dominant inheritance of NF-1 and that JCS is just a special subtype of NF-1 as suggested by Colby et al. and Baumhauer et al. [9, 10].

### Genetic basis

Baumhoer et al. performed DNA sequencing on 59 patients with non-ossifying fibromas (NOFs). They identified mutations in three genes: KRAS, FGFR1, and NF1. Specifically, KRAS mutations were found in 64% of patients, suggesting somatic mutations due to the lethality of germline KRAS mutations. FGFR1 mutations were detected in 14% of patients, also considered somatic as the germline mutations are associated with osteoglophonic dysplasia. NF1 mutations were observed in 2 cases (3.39%). Polyostotic NOFs, which are rarer, showed an association with neurofibromatosis type 1 (NF1) and JCS [11].

Stewart et al. studied 14 JCS cases and found somatic NF-1 mutations in 13 of them. These 13 cases met the NIH criteria for neurofibromatosis-1 [12]. Colby emphasized the correlation between neurofibromatosis-1 and multiple NOFs, recommending radiographic surveys [9].

### Management strategies

Being most commonly occurring in the distal femur and proximal tibia, Colby and Saul suggested doing radiographs of both knees for patients suspected to have JCS [4]. However, they can be located in other regions of the upper limbs and diaphysis of long bones. Thus, a skeletal survey is recommended to spot all missed and skip lesions. Being more susceptible to pathological fractures, Jaffe Campanacci patients with NOF in the distal femur are better managed operatively by prophylactic fixation according to many authors [3, 6, 8]. Campanacci et al. suggested that more than half of the cases suffer at least one fracture throughout their lifetime [3]. Hence, we recommend that diagnosed patients engage in proactive strategies to avoid trauma, thereby reducing the likelihood of fractures.

Hau et al. reported a case of a fifteen-year-old patient with JCS when excision, curettage, allograft strut-grafting, and plate fixation were done. Healing occurred six months post-operatively [13]. Chen Yang et al. performed intralesional excision and allograft grafting, but, instead of rigid fixation, they adopted restricted weight-bearing for three weeks. Bone union was achieved after six months [14]. These findings suggest that healing at the sites of the bone lesions was not impaired. In our case, we adopted open reduction and internal fixation without grafting. Complete bone union was achieved after 6 months.

JCS is not the only syndrome overlapping with NF-1. Some other syndromes overlap with NF-1 as Neuro-cardio-facial dysmorphism which is characterized by mental retardation or cognitive dysfunction, congenital heart defects, facial dysmorphism [15], and legius syndrome which is characterized by CALMS, macrocephaly, skin freckles, cognitive disorder and short stature [16].

Patient and family education plays a vital role by explaining the benign nature of NOFs, the associated conditions and the need for regular follow-up. Radiographic findings should be described to patients and their families, along with instructions on symptom recognition and pain management. Genetic counseling is essential, as it may be passed on to children as shown in this study. Lastly, psychosocial support acknowledges the emotional impact of chronic conditions. This can be helped by connecting patients with support groups and counseling.

### Literature review

**Fig. 9:**
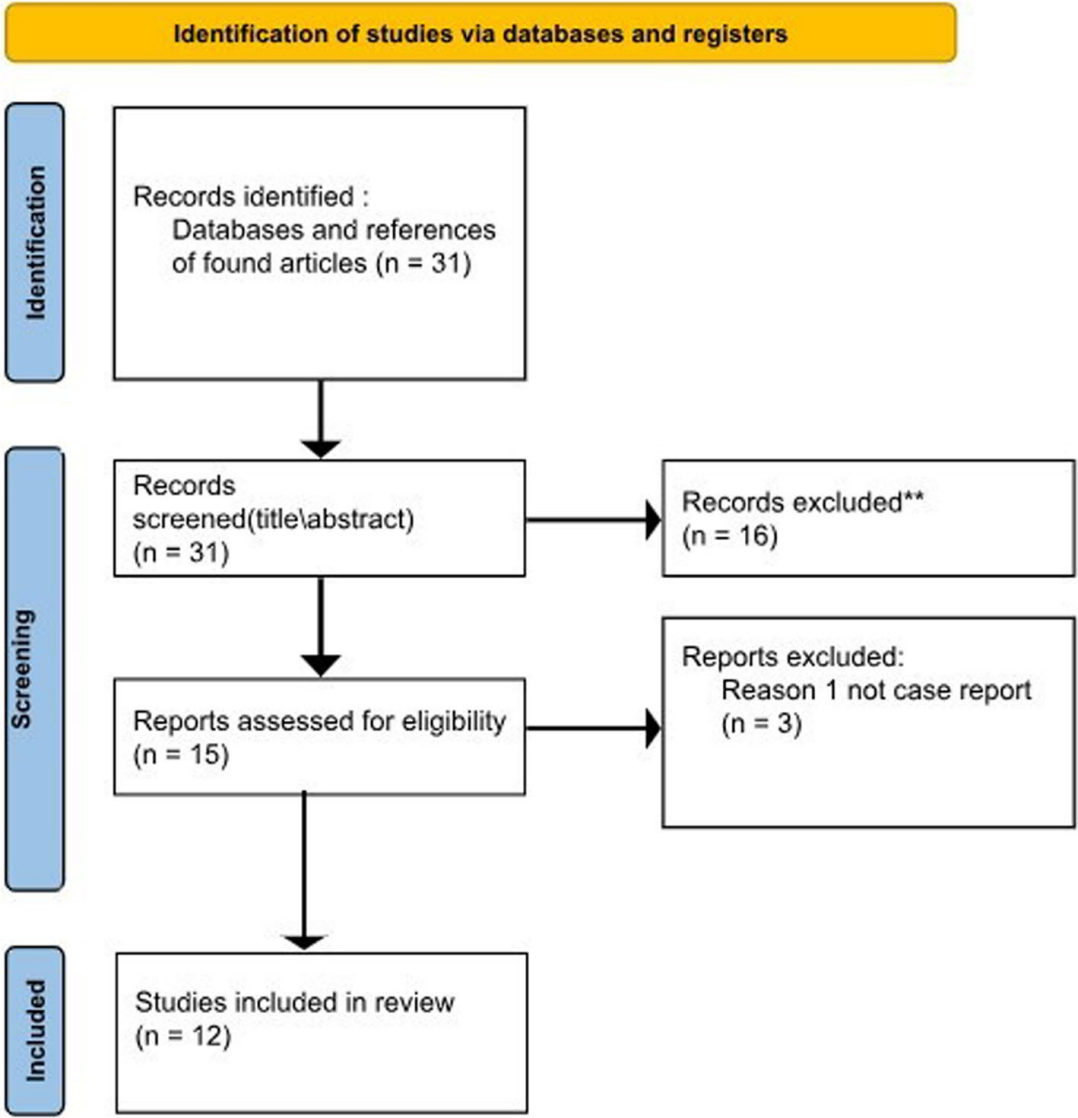
Prisma chart of the included studies

Authors searched literature using many literature databases including Medline, the web of science, Google Scholar, and the Egyptian knowledge bank databases. All relevant papers had their references searched as well for related articles. The present review encloses all case reports on Jaffe-Campanacci syndrome. A total of 31 unique studies from all databases were identified. English-language articles that reported case reports and series were included, which narrowed down the included articles to 12 (Fig. 9). Details of the cases are listed below in Table 1.

**Table 1:** List of Jaffe–Campanacci syndrome cases reported in the literature

Case number	Author and publication date	Gender	Age at diagnosis	Family history of JCS present	Presence of a fracture and presenting features	Diagnosed with neurofibromatosis
Case 1	M. Campanacci, 1983 [3]:	Male	11	N/A	Four pathological fractures; tibial osteotomy for valgus deformity of the knee	No Previous diagnosis
Case 2	M. Campanacci, 1983 [3]:	Female	6	N/A	One pathological fracture	No Previous diagnosis
Case 3	M. Campanacci, 1983 [3]:	Male	17	N/A	-	No Previous diagnosis
Case 4	M. Campanacci, 1983 [3]:	Male	12	N/A	Two pathological fractures	No Previous diagnosis
Case 5	M. Campanacci, 1983 [3]:	Male	11	N/A	Two pathological fractures	No Previous diagnosis
Case 6	M. Campanacci, 1983 [3]:	Female	5	N/A	One pathological fracture	No Previous diagnosis
Case 7	M. Campanacci, 1983 [3]:	Female	18	N/A	-	No Previous diagnosis
Case 8	M. Campanacci, 1983 [3]:	Male	12	N/A	-	No Previous diagnosis
Case 9	M. Campanacci, 1983 [3]:	Male	14	N/A	One pathological fracture	No Previous diagnosis
Case 10	M. Campanacci, 1983 [3]:	Male	6	N/A	One pathological fracture	No Previous diagnosis
Case 11	D.C. Howlet, 1998 [17]:	Male	18	Sister and Mother had NF1	pathological fracture is seen involving a lesion in the lower tibia,non-ossifying fibromas, and shin pain	Previous Diagnosis
Case 12	D.C. Howlet, 1998 [17]:	Female	15	Brother and Mother had NF1	multiple non-ossifying fibromas was made on the radiological findings	No Previous diagnosis
Case 13	R. S. Colby, 2003 [9]:	Female	13	Negative NF1 and bone lesions	Fracture left femur at 11 years. Presnted with pain over the fracture site. Multiple NOF	Previous Diagnosis
Case 14	R. S. Colby, 2003 [9]:	Male	15	Negative NF1 and bone lesions	Fracture femurs at age 14. Multiple NOF	Previous Diagnosis
Case 15	R. S. Colby, 2003 [9]:	Male	13	Negative NF1 and bone lesions	Leg problems and multiple NOF	Previous Diagnosis
Case 16	R. S. Colby, 2003 [9]:	Male	17	Negative NF1 and bone lesions	Leg problems and multiple NOF	Previous Diagnosis
Case 17	Ammar C. Al-Rikabi, 2005 [4]	Male	6	N/A	Right upper tibial lytic bone lesion	Previous Diagnosis
Case 18	Mehmet Sonar, 2012 [18]	Male	13	N/A	pathological fracture of the distal part of the left tibia	No Previous diagnosis
Case 19	Chen Yang, 2012 [14]	Female	10	Negative for NF1	Fracture of the humerus and impending. fracture in the right femur Multiple osteolytic lesions	Previous Diagnosis
Case 20	Adnan Sevencan, 2013 [19]	Male	10	N/A	Lesion in the proximal part of the left humerus	No Previous diagnosis
Case 21	Stéphane Cherix, 2014 [20]	Female	17	Father had NF1	Fracture of her right femur	Previous Diagnosis
Case 22	Eun mi Choi, 2016 [21]	Female	9	Negative for NF1	Multiple NOF	No Previous diagnosis
Case 23	Alessandro Corsi, 2017 [22]	Male	18	N/A	A fracture in the radius was detected, and multiple lytic lesions at various ages	No Previous diagnosis with NF1, but was diagnosed with an aneurysmal bone cyst
Case 24	Yong Han, 2019 [23]	Female	11	Negative for NF1	Multiple lesions and nonossifying fibromas	Previous Diagnosis
Case 25	Mohsen Qutbi, 2019 [2]	Male	27	N/A	Fracture with an underlying nonossifying fibromas	No Previous diagnosis
Case 26	Silvia Vannelli, 2020 [24]	Male	20	N/A	Fractures and lesions at various stages of life	Previous Diagnosis
Case 27	Rajaa Bousmara, 2023 [25]	Male	11	Negative for NF1	No fractures, Multiple CALM lesions, NOF’s and Lisch nodules	No Previous diagnosis

Eighten of the reported 27 cases were males (66.667%). Out of the 27 reported cases, only 11 had been reported if they had a family history of similar lesions or NF-1 or not. 3 cases (37.5%) were found to have a positive family history where at least one parent had either JCS or NF-1. The majority of cases, 17 (62.96%) out of 27 cases, had their first presentation as a pathological fracture. This could be because most cases remain undiagnosed as JCS and don’t do radiographs until they suffer from a fracture.

### Complications and long term outcomes

Despite a high association of pathological fractures over the NOFs [3], these lesions tend to regress as the patient reaches skeletal bone maturity [12]. Giant cell granulomas tend to appear in the second to third decade of life and may cause facial asymmetry and dental malocclusion. There is a documented association with systemic abnormalities which includes cryptorchidism, hypogonadism, ocular abnormalities, cardiovascular abnormalities and intellectual disabilities [18]. Multidisciplinary management is essential for addressing the orthopedic, dental and systemic challenges associated with this syndrome.

### Future directions

For future research directions in the study of JCS, it’s essential to explore the genetic basis of the condition further. This includes investigating the potential hereditary nature of the syndrome through a comprehensive familial analysis and history.

## Conclusion

JCS is characterized by its multiple non-ossifying fibromas, Cafe-au-lait macules, and axillary/inguinal freckling and usually is accompanied by symptoms of NF-1. This case series suggested that JCS may be a variety of NF-1 as it shows there may be autosomal dominant transmission similar to NF-1 along with its manifestations. These cases are likely to present with at least one fracture on top of NOF lesions and hence need evaluation follow-up and assessment of cortical thinning over NOF especially in high weight-bearing bones such as the femur and tibia.

## Data Availability

Data is provided within the manuscript and presented on request. The data regarding the studies included in the systematic review is present as part of the manuscript and is present in Table 1.

## Abbreviations

CALM: Café-au-lait macule
FGFR1: Fibroblast growth factor receptor 1
JCS: Jaffe-Campanacci syndrome
KRAS: Kirsten rat sarcoma virus
MRI: Magnetic resonance imaging
N/A: Not available
NF-1: Neurofibromatosis-1
NOF: Non-ossifying Fibroma
WHO: World Health Organization
